# Development and Validation of a Necroptosis-Related Prognostic Model in Head and Neck Squamous Cell Carcinoma

**DOI:** 10.1155/2022/8402568

**Published:** 2022-02-18

**Authors:** Zhijing Zhang, Xinglin Hu, Dan Qiu, Yuchen Sun, Lei Lei

**Affiliations:** Department of Histology and Embryology, Harbin Medical University, Harbin, Heilongjiang Province, China

## Abstract

Necroptosis is a new regulated cell-death mechanism that plays a critical role in various cancers. However, few studies have considered necroptosis-related genes (NRGs) as prognostic indexes for cancer. As one of the most common cancers in the world, head and neck squamous cell carcinoma (HNSCC) lacks effective diagnostic strategies at present. Hence, a series of novel prognostic indexes are required to support clinical diagnosis. Recently, many studies have confirmed that necroptosis was a key regulated mechanism in HNSCC, but no systematic study has ever studied the correlation between necroptosis-related signatures and the prognosis of HNSCC. Thus, in the current study, we aimed to construct a risk model of necroptosis-related signatures for HNSCC. We acquired 159 NRGs from the Kyoto Encyclopedia of Genes and Genomes (KEGG) and compared them with samples of normal tissue downloaded from The Cancer Genome Atlas (TCGA), ultimately screening 38 differentially expressed NRGs (DE-NRGs). Then, by Cox regression analysis, we successfully identified 7 NRGs as prognostic factors. We next separated patients into high- and low-risk groups via the prognostic model consisting of 7 NRGs. Individuals in the high-risk group had much shorter overall survival (OS) times than their counterparts. Furthermore, using Cox regression analysis, we confirmed the necroptosis-related prognostic model to be an independent prognostic factor for HNSCC. Receiver operating characteristic (ROC) curve analysis proved the predictive ability of this model. Finally, Gene Expression Omnibus (GEO) data sets (GSE65858, GSE4163) were used as independent databases to verify the model's predictive ability, and similar results obtained from two data sets confirmed our conclusion. Collectively, in this study, we first referred to necroptosis-related signatures as an independent prognostic model for cancer via bioinformatics measures, and the necroptosis-related prognostic model we constructed could precisely forecast the OS time of patients with HNSCC. Utilizing the model may significantly improve the diagnostic rate and provide a series of new targets for treatment in the future.

## 1. Introduction

An uncontrollable form of cell death, accidental cell death (ACD), can be provoked by exposure to harmful microenvironmental conditions (e.g., high temperatures, oxygen deficiency, and external force) [1]. However, cell death can also be regulated when homeostasis perturbations are mild; this is known as regulated cell death (RCD), and apoptosis is considered the main form of RCD [2]. According to traditional beliefs, necrosis has several morphological features (e.g., cytoplasmic swelling and loss of plasma membrane integrity) present during ACD progression, so necrosis is commonly regarded as a type of passive cell death that always occurs in ACD [3, 4]. However, in these years, accumulating studies have found there some nonapoptotic cell deaths occur with partial or complete necrotic morphology in RCD, such as necroptosis, ferroptosis, and pyroptosis [3, 5]. For example, swelling and disrupted membranes were observed morphologically among cells undergoing necroptosis, which are typical features of ACD [6]. In addition, many diseases are known to be correlated with these nonapoptotic cell deaths, including inflammatory, cardiovascular, and neurological diseases [4]. Thus, these nonapoptotic cell deaths were considered as an example series of a new type of RCD and have received increasing attention.

Among these nonapoptotic cell deaths, necroptosis is a recently discovered regulation form of necrosis mediated by protein kinase C-related kinases (e.g., PRK1) as well as receptor-interacting serine-threonine kinase 3 (RIPK3), which can assemble into the necrosome to initiate necroptosis [7–9]. With the help of executioner protein mixed-lineage kinase-like (MLKL), necroptotic cells can permeabilize cell membranes and promote the release of intracellular contents [9]. There is an apparent relationship between necroptosis and prognosis in many cancers. In a study of >60 tumors, the expression of RIPK3 was extremely decreased, and patients with lower levels of RIPK3 had a worse prognosis [10, 11]. By contrast, necroptosis may also be a promoter of various tumors. Some studies have found that many cancer cells can induce endothelial cell necroptosis to promote extravasation and metastasis [12]. Ando et al. demonstrated that, in pancreatic cancer, by upregulating C-X-C motif chemokine 5 (CXCL5) and C-X-C-motif chemokine receptor-2 (CXCR2), cancer cells can enhance migration and invasion by inducing necroptosis [13]. Collectively, these studies suggest that necroptosis is an indispensable mechanism with complicated biological functions in tumorigenesis and tumor invasion. However, although necroptosis is considered a key mechanism in many cancers, few studies have considered necroptosis as an independent prognostic indicator.

Until 2018, HNSCC had been the sixth most common cancer worldwide, with a mortality rate that ranked eighth among all cancers [14]. Nearly 890,000 individuals were diagnosed with HNSCC and 450,000 deaths from HNSCC occurred in 2018 worldwide [15]. HNSCC is the most common malignancy to arise in the head and neck, and almost all HNSCCs originate from the mucosal epithelium in the oral cavity, pharynx, and larynx [16]. The onset of HNSCC is mainly related to exposure to tobacco-derived carcinogens, excessive alcohol consumption, and human papillomavirus infection [16]. Despite several recently developed inspiring therapeutic strategies, the low survival rates (about 40%–50%) and few effective indexes for monitoring cancer development remain primary problems that need to be resolved [17]. Furthermore, necrosis has already been considered a promising prognostic factor for many solid tumors, such as colorectal cancer [18], but it remains unclear whether necroptosis is an effective prognostic factor. Moreover, necroptosis has been suggested to be relevant to many HNSCC clinicopathological features [19], but no systematic studies to date have explored the association between necroptosis-related signatures and HNSCC prognosis. Therefore, we systematically evaluated the RNA-sequencing and clinical data to establish and validate a necroptosis-related prognostic model and then take advantage of the model to predict individual prognoses for HNSCC patients.

## 2. Materials and Methods

### 2.1. Human Necroptosis-Related Gene (NRG) Set

A total of 159 genes were collected from the Kyoto Encyclopedia of Genes and Genomes (KEGG) (https://www.kegg.jp/). The complete NRGs are listed in Table S1.

### 2.2. Data Acquisition and Preprocessing

The Cancer Genome Atlas (TCGA) database (https://portal.gdc.cancer.gov/) was used to obtain RNA-sequencing (RNA-seq) data about HNSCC. In total, we obtained 528 clinical and 546 RNA-seq data points. We excluded samples whose follow-up time was <30 days. Two independent data sets were downloaded from the GEO database (accession nos. GSE65858 and GSE4163). Log_2_ transformation was utilized to standardize the total RNA-expression data.

### 2.3. Differentially Expressed mRNA (DEG) Analysis and Functional Analysis

The R programming language (version 4.1.0) was utilized to perform a series of bioinformatics analyses. Differentially expressed (DE)-NRGs were screened using the R package limma. To identify biological functions, we carried out various functional enrichment analyses, including KEGG, Gene Ontology (GO), protein-protein interaction (PPI), and gene set enrichment analysis (GSEA) analyses. The enrichment plots were completed using the GOplot package.

### 2.4. Establishment of the Prognostic Model

Using a univariate Cox regression analysis, we recognized the candidate NRGs associated with prognosis. Furthermore, a multivariate Cox regression analysis was implemented to evaluate NRGs screened by the univariate Cox regression analysis. Seven NRGs were successfully identified as the prognostic indexes contributing to the prognosis of HNSCC. Based on the expression of each NRG multiplied by a regression coefficient (*β*) using the equation risk score = ∑_**i**=**1**_^**n**^*β*_**i**_*∗*(expression **of** **NRGs**_**i**_), we determined the risk score for each patient. Then, all patients were classified into two risk groups using the median risk score. Via log-rank statistical methods and Kaplan–Meier survival analysis, we detected the different OS times of the two risk groups. The predictivity of the model was also assessed via Cox regression analysis. Finally, we determined the accuracy of this prognostic model via time-related receiver operating characteristic (ROC) curve analysis.

### 2.5. Online Databases Verification

A series of online databases were used to verify this prognostic model. The TIMER database (https://timer.cistrome.org/) was utilized to analyze the differential expression of NRGs involved in our model; Human Protein Atlas (HPA, https://www.proteinatlas.org/) was used to assess the NRGs protein expression in tumor and nontumor tissues; and the cBioPortal database (https://www.cbioportal.org/) was performed to check gene alteration of the NRGs.

### 2.6. Statistical Analysis

The R programming language (version 4.1.0) was utilized to perform data analysis. *P* < 0.05 was identified to indicate statistical significance. Key NRGs associated with survival were selected using univariate and multivariate Cox analyses. Kaplan–Meier survival analysis with log-rank statistical methods was conducted to compare OS time and plot survival curves. The predictive ability of this model was testified using ROC and area under the ROC curve (AUC) analyses.

## 3. Results

### 3.1. Identification of 38 DE-NRGs in HNSCC

**Figure 1:**
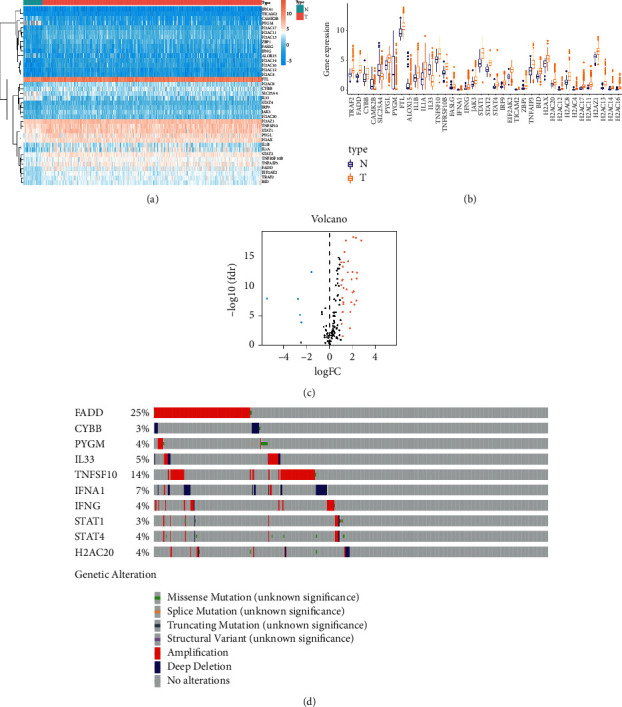
The differentially expressed necroptosis-related genes (DE-NRGs). (a) A heatmap showing the expression of the DE-NRGs. (b) A boxplot indicating the expression of the DE-NRGs. (c) A volcano plot showing the expression of the DE-NRGs. (d) Mutation profile of the DE-NRGs; the mutation rate of the total 10 genes was ≥3%.

Using the TCGA HNSCC data set, we collected RNA-seq data, including 502 HNSCC and 44 normal tissues; subsequently, 495 clinical data points of HNSCC patients (follow-up period was ≥30 days) were included in this study. The NRG list was downloaded from the KEGG. Compared with the normal samples, we obtained 38 DEG-NRGs (false discovery rate (FDR) <0.05 and log2|fold change|(log2 FC) >1), including 5 downregulating genes (PYGM, SLC25A4, CAMK2B, ALOX15, IL33) and 33 upregulating genes (H2AZ1, H2AX, CYBB, FTL, TICAM2, TNFAIP3, TRAF2, H2AC20, FASLG, TNFRSF10B, JAK3, TNFSF10, PYGL, BID, STAT2, EIF2AK2, STAT4, STAT1, IRF9, IFNA1, IL1B, H2AC4, H2AC8, ZBP1, H2AC17, FADD, H2AC12, IFNG, H2AC11, IL1A, H2AC14, H2AC16, H2AC13). The expression profile of these DEGs is exhibited in a heatmap, boxplot, and volcano plot (Figures 1(a)–1(c)) separately. Then, we checked the genetic mutation of these DE-NRGs using the cBioPortal database. As shown in Figure 1(d), we found 10 genes whose mutation rate was ≥3% among all DEGs (The gene alteration of all DEGs in Figure S1); among these, “amplification” and “deep deletion” were the most common types of genetic alterations.

### 3.2. Functional Analysis of the DE-NRGs

**Figure 2:**
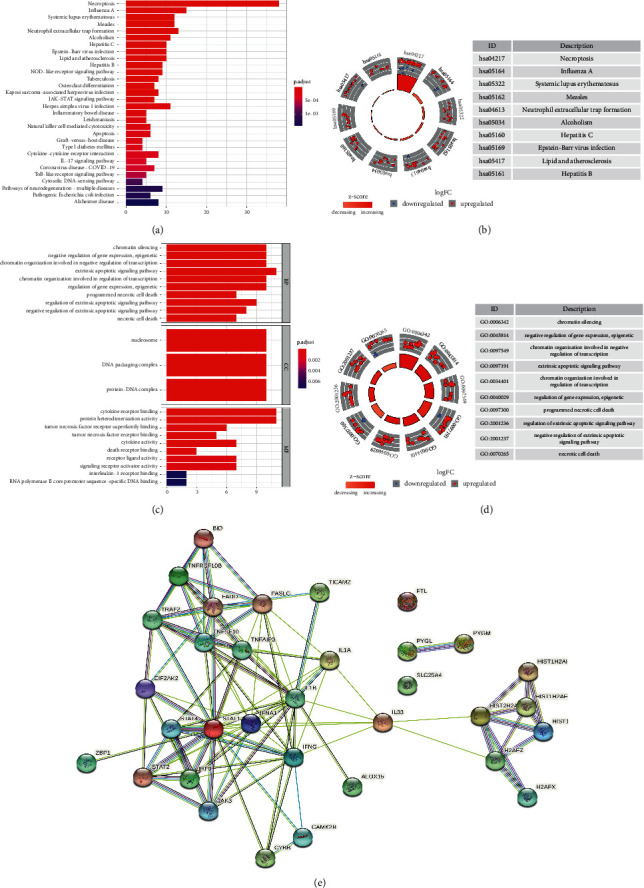
Function analysis of differentially expressed necroptosis-related genes (DE-NRGs). (a, b) Histogram and circle about the Kyoto Encyclopedia of Genes and Genomes enrichment of the top 30 significant DE-NRGs. (c, d) Histogram and circle about gene ontology enrichment of the top 30 significant DE-NRGs. (e) Protein-protein interaction network of the protein transcribed by 33 DE-NRGs.

To figure out the detailed biological functions of these genes, 38 DE-NRGs were incorporated into a functional enrichment analysis. We illustrated the top 30 results of KEGG and GO enrichment analyses (Figures 2(a)–2(d)). As shown in Figures 2(a) and 2(b), KEGG enrichment indicated that the DE-NRGs were mainly involved in the following pathways: (1) JAK-STAT signaling pathway; (2) diseases involving necroptosis, such as influenza A and systemic lupus erythematosus; and the (3) NOD-like receptor signaling pathway. Of note, the coronavirus disease 2019 (COVID-19) pathway was also enriched by the aforementioned DE-NRGs. Recent studies have suggested necroptosis may also be a promising factor in COVID-19 [4, 20]. According to our results, it is reasonable to presume that some similar mechanisms of necroptosis probably exist in both HNSCC and COVID-19. In the GO enrichment analysis (Figures 2(c) and 2(d)), the biological processes of DE-NRGs mainly included apoptosis, necrosis, chromatin silencing, and epigenetic regulation. Furthermore, to explore the interaction among the proteins transcribed by these DE-NRGs, we analyzed the PPI network using the STRING database. In total, 33 proteins were involved in the network, and the gene annotations, as well as combined scores, are summarized in Figure 2(e) and Table S2.

### 3.3. Constructing a Model of Necroptosis-Related Signatures

**Figure 3:**
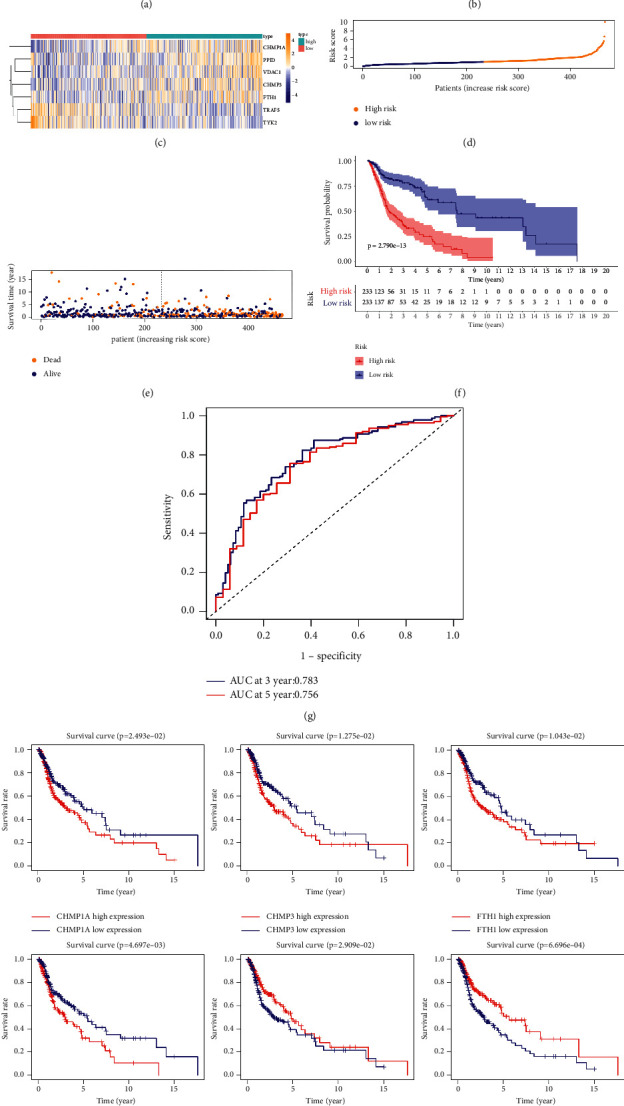
Establishing a necroptosis-related prognostic model. (a) A univariate Cox analysis of NRGs is illustrated by a forest plot. (b) A forest plot illustrates the multivariate Cox analysis of the NRGs from (a). (c) A heatmap of 7 NRGs showing the different expressions between the two risk groups. (d) The risk scores for HNSCC patient are plotted in ascending order. (f) Different OS times between the two risk groups were demonstrated using Kaplan–Meier analysis. (g) The receiver operating characteristic curve analysis in the Cancer Genome Atlas head and neck squamous cell carcinoma cohort. (h) Kaplan–Meier curves of single necroptosis-related genes.

Using a univariate Cox analysis, we screened 15 NRGs with prognostic value (*P* < 0.05) (Figure 3(a) and Table S3). Then, a multivariate Cox regression analysis was taken advantage to evaluate these NRGs, and 7 NRGs were finally identified as significantly associated with prognosis (Figure 3(b)). Among them, two genes (TRAF5 and TYK2) were protective factors, and the others (PPID, VDAC1, FTH1, CHMP3, and CHMP1A) were harmful factors (Figure 3(h) and Table 1). Then, we constructed a necroptosis-related prognostic model using the following formula involving the 7 genes: risk score = (−0.31157 *∗* TRAF5) + (0.094121 *∗* PPID) + (0.009295 *∗* VDAC1) + (0.002142 *∗* FTH1) + (0.054985 *∗* CHMP3) + (0.009986 *∗* CHMP1A) + (−0.06185 *∗* TYK2). Using the median risk score (median risk score = 1.027), we separated patients into high-risk (*n* = 233) and low-risk (*n* = 233) groups (Figure 3(d)). As illustrated in Figure 3(c), the expression of protective factors was extremely increased in the low-risk group compared with the high-risk group. In contrast, in the high-risk group, that of harmful factors was strikingly increased (Figure 3(c)).

There were also several significant differences in survival status and OS time between the two risk groups. A higher risk score was present among dead patients, while those that remained alive had lower risk scores (Figure 3(e)). In addition, compared with the patients with higher risk scores, a much longer OS time was discovered among patients with a lower risk score (Figure 3(f)). We carried out ROC curve analysis for 3- and 5-year OS times to estimate the prognostic power of the model, and the AUCs of the ROC curve analysis were calculated to be 0.783 and 0.756, respectively (Figure 3(g)). These results preliminarily indicated that it is robust to predict the outcome of HNSCC patients.

### 3.4. The Prognostic Model Consisting of 7 NRGs Is an Independent Prognostic Index

**Figure 4:**
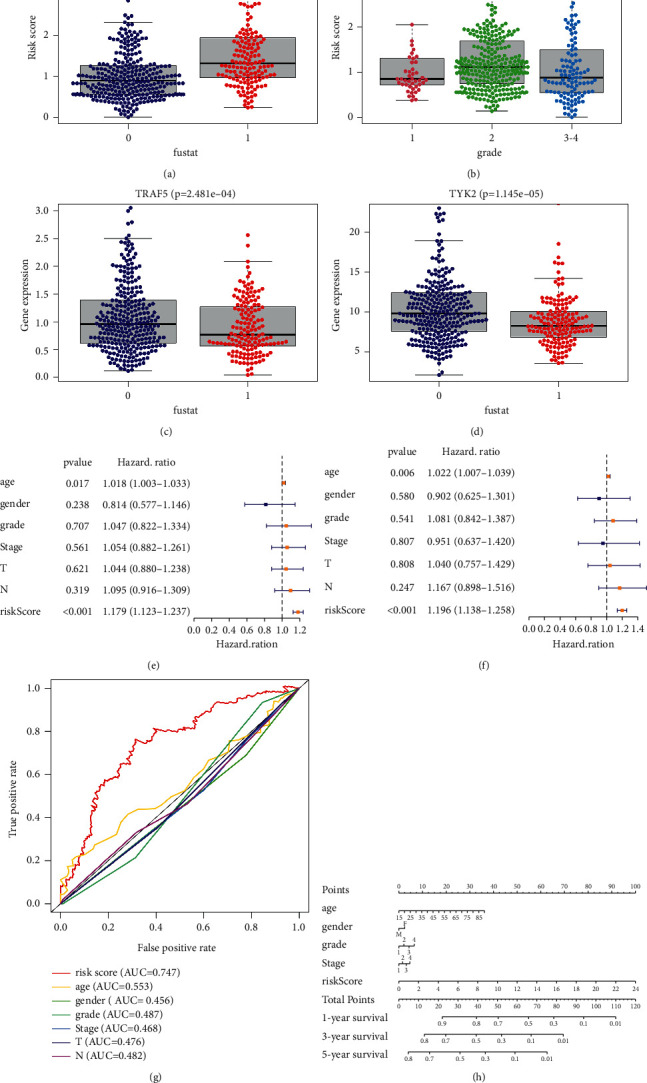
Prognostic model constructed by the necroptosis-related genes (NRGs) showing an excellent predictive ability. (a, b) The correlation between risk score and survival status and grade. (c, d) The correlation between single NRGs and survival status. (e, f) Risk scores and clinicopathological characteristics were determined by Cox regression analysis. (g) Receiver operating characteristic curve analysis at 5-year survival rate testified the precision of this model. (h) The nomogram indicated 5 characteristics in The Cancer Genome Atlas as follows: age, gender, grade, stage, and risk score.

Since the risk model had accurate predictivity, we wondered whether this model was an independent prognostic index. Hence, several clinicopathological characteristics and risk scores were entered into a correlation analysis. As there was no striking difference in tumor *M* stage among almost all patients, the *M* stage was excluded from our analysis. Results suggested deceased patients (*P* < 0.001) and patients with high-grade tumors (*P*=0.01) had higher risk scores (Figures 4(a) and 4(b)). This meant that the expression of NRGs constituting our model may significantly influence tumor malignancy and survival status. Furthermore, the single NRGs (CHMP1A, CHMP3, FTH1, TRAF5, TYK2, VDAC1, and PPID) were associated with some clinicopathological parameters as well; specifically, TRAF5, TYK2, VDAC1, FTH1, and CHMP1A were correlated with the survival status, while TRAF5, CHMP3, FTH1, PPID, TRAF, and TYK2 showed great correlation with the tumor grade (Figures 4(c), 4(d), and S2). Then, to confirm the model as an independent predictor of HNSCC, Cox regression analysis was conducted. Our results suggested that “risk score” and “age” were candidate variables related to the OS time by univariate Cox regression analysis (Figure 4(e)). After that, we performed a multivariate Cox analysis to assess these variables, and the risk score was successfully identified as an independent prognostic index (*P* < 0.001) (Figure 4(f)). The AUC for the risk score was 0.747 (Figure 4(g)). Finally, using 4 clinicopathological parameters and the risk score, a nomogram was illustrated to predict the survival rates (Figure 4(h)). In summary, these results confirmed the necroptosis-related prognostic model as an independent prognostic index of clinical characteristics.

### 3.5. Validation of the Necroptosis-Related Prognostic Model via Two Independent GEO Data Sets

**Figure 5:**
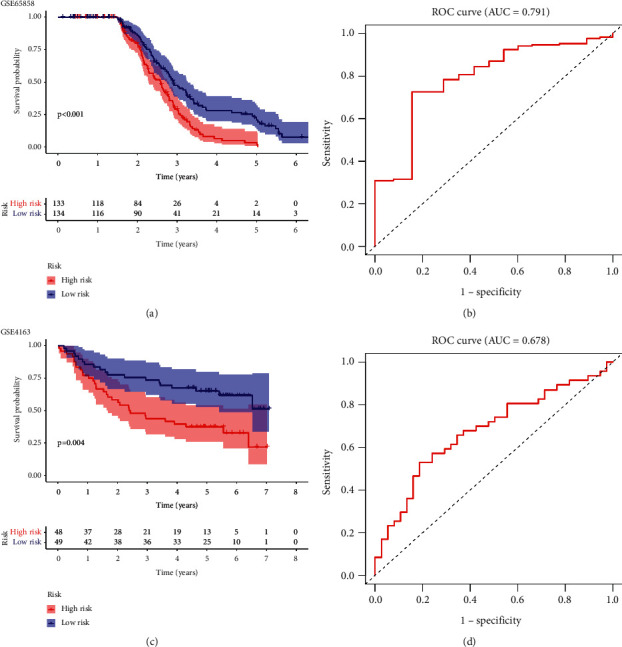
Validation of the risk model via two independent databases. (a, c) The different overall survival (OS) time between two risk groups based on the GSE65858 and GSE4163data sets. (b, d) Receiver operating characteristic curve (ROC) analysis based on the GSE65858 and GSE4163 data sets.

To examine whether the model had excellent stability and reproducibility, we conducted external validation using the GEO data sets (accession nos. GSE65858 and GSE4163). Using the median risk score, we employed the same formula obtained from the TCGA-HNSCC data set to separate patients into two risk groups. Consistent with the TCGA-HNSCC data set, regardless of whether considering GSE65858 or GSE4163, a much longer OS time was observed in the patients in the low-risk group compared with those in the high-risk group (Figures 5(a) and 5(c)). In GSE65858 and GSE4163, the ROCs of the 5-year survival rate were 0.791 and 0.678, respectively (Figures 5(b) and 5(d)). In conclusion, by using the aforementioned 2 GEO data sets, we verified the generality of our prognostic model, and these results are similar to those obtained from the TCGA-HNSCC data set, demonstrating the predictive ability and accuracy of the model.

#### 3.5.1. GSEA

**Figure 6:**
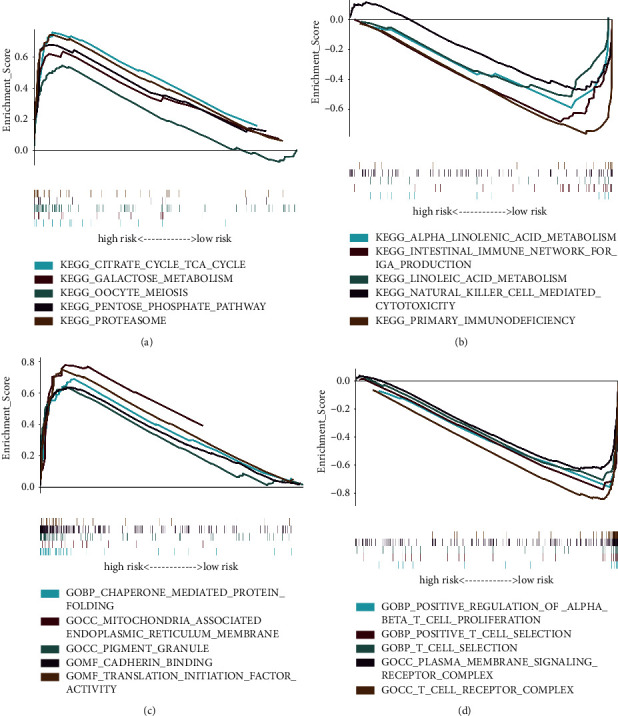
Gene set enrichment analysis of the necroptosis-related prognostic model in head and neck squamous cell carcinoma (HNSCC). (a, b) Top 5 Kyoto Encyclopedia of Genes and Genomes enrichment results of the high- and low-risk groups in HNSCC. (c, d) Top 5 gene ontology enrichment results of the high- and low-risk groups in HNSCC.

We further executed GSEA to evaluate the necroptosis-related pathways in the TCGA-HNSCC data set. In total, we recognized 178 KEGG pathways (Tables S4 and S5) and 5,391 GO terms (Tables S6 and S7).  Figure 6 illustrates the top 5 KEGG pathways and GO terms of the two risk groups. In the TCGA-HNSCC data set, we revealed that many metabolism-related pathways were activated in the high-risk group, such as tricarboxylic acid cycle (TCA cycle) pathway, galactose metabolism, and pentose phosphate pathway (Figure 6(a)). This suggested that some critical metabolic processes might be altered when necroptosis is occurring. In addition, according to the GO enrichment results, several biological processes related to protein folding were also enhanced in the high-risk group (Figure 6(b) and Table S5). In the low-risk group, KEGG and GO enrichment results uncovered that the genes were mostly connected with some immune-related pathways. In summary, we concluded that necroptosis may effectively enhance metabolism, accelerate the cell cycle, and increase biosynthesis.

### 3.6. Online Database Analysis

**Figure 7:**
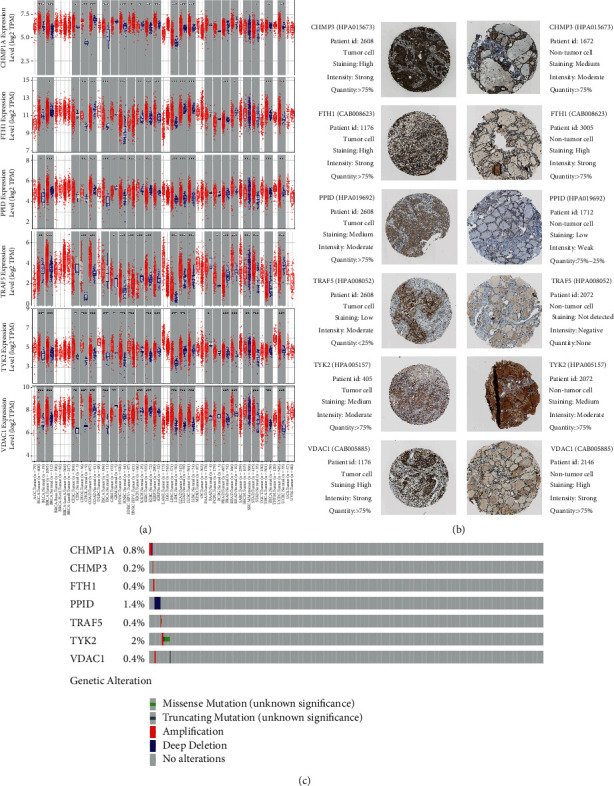
Online database analysis. (a) The TIMER database illustrated the differential expression of 6 necroptosis-related genes (NRGs) involved in our model (no CHMP3 data are available in the TIMER database). (b) The HPA database showed the protein expression of 6 NRGs involved in our model (no CHMP1A data are available in the HPA database). (c) The gene alterations of 7 NRGs involved in our model were explored using the cBioPortal database.

To validate our model, we checked it against various online databases. First, we explored the differential expression of 7 NRGs in our model using the TIMER database, which showed that 6 NRGs (there are no CHMP3 data available in the TIMER database) were significantly overexpressed in HNSCC (Figure 7(a)). Furthermore, the HPA database was also utilized to analyze the NRGs. In agreement with our results, the HPA database confirmed that TRAF and TYK2 were protective factors in HNSCC, and the remaining genes were harmful factors (data not shown). Considering that HNSCC usually occurs in the thyroid, and the HPA database does not list normal data of the head and neck separately, we used normal thyroid tissue as the control. The immunohistochemistry (IHC) data of CHMP3, FTH1, PPID, TRAF5, TYK2, and VDAC1 (no CHMP1A protein-expression data are available in HPA) were acquired from HPA, as illustrated in Figure 7(b). Finally, using the cBioPortal database, we explored the gene alterations of the 7 NRGs. The cBioPortal database uncovered that there was no striking mutation in all 7 NRGs in HNSCC (Figure 7(c)). It suggested that gene mutation may not be the main reason for the NRG overexpression observed in the TIMER data set; instead, the abnormal expression was more likely caused by dysfunctional regulatory pathways.

## 4. Discussion

Necroptosis has been identified as a new form of RCD with necrotic-like features. Various special features in necroptosis differ from those in apoptosis, such as membrane permeabilization, releasing damage-associated molecular patterns, and cell swelling [21]. However, necroptosis is also a “double-edged sword.” Importantly, the protective effectiveness of necroptosis can maintain homeostasis and trigger powerful antitumor immunity, while, on the other hand, necroptosis can be a tumor-driver facilitating invasion and migration [19, 22]. In HNSCC, necrosis has been confirmed as a common pathological characteristic [23]. Many researchers have found that necrosis can effectively promote tumor invasion and progression via inducing hypoxia inside the tumor [18, 24]. Recently, Li et al. demonstrated that about 50% of necrosis in HNSCC is caused by necroptosis and suggested that necroptosis can release damage-associated molecular patterns to enhance the migration and invasion of HNSCC cells [19]. Hence, the necrosis observed in HNSCC tumors may not simply be caused by ACD, and the necroptosis, also causing necrosis, is perhaps another important phenomenon at play during HNSCC development.

Although many studies have argued connections between NRG and HNSCC, there are no systematic studies considering necroptosis-related signatures as a prognostic index to forecast the prognosis of HNSCC patients. As the field of bioinformatics develops, accumulating novel measures can provide effective tools to explore gene signatures. Hence, by utilizing the TCGA-HNSC data set, we firstly identified 38 differentially expressed NRGs and then incorporated them into KEGG, GO, and PPI analyses. The results indicated these genes are mostly enriched in necroptosis-related disease, regulation pathways of necroptosis and apoptosis, and other processes associated with necroptosis. Unexpectedly, the DE-NRGs in HNSCC were also enriched in the COVID-19 pathway. Recently, some studies have suggested necroptosis is a promising factor that plays a vital role in COVID-19 [4, 20]. Therefore, we deduced that some similar necroptosis-related mechanisms may exist in both HNSCC and COVID-19. Future studies focusing on exploring this potential connection may provide some new targets for COVID-19 clinical treatment.

Furthermore, to identify HNSCC prognosis-related NRGs, we carried out Cox regression analyses. Seven NRGs manifested outstanding correlations with the OS time of HNSCC and were utilized to establish prognostic models. According to the median risk score, HNSCC patients were separated into two groups. Compared with the high-risk group, patients in the low-risk group had longer OS times. A necroptosis-related risk model was also confirmed as an independent prognostic index. In addition, we found the risk score was extremely related to survival status and tumor grade. In conclusion, these results demonstrated that our necroptosis-related prognostic model had a great ability to predict the pathogenesis and progression of HNSCC; moreover, it was also highly correlated with OS time.

Previous studies have confirmed some NRGs involved in our risk model are indispensable in HNSCC. As the regulatory gene with the smallest hazard ratio (HR) (0.732) in our study, TAR5 has been studied in many cancers. Previous studies have demonstrated that TAR5 is a key target to inhibit tumor cell migration or proliferation, and the expression of TRAF negatively correlates with prognosis [25–27]. However, in this study, we obtained the opposed conclusion that TAR5 is a protective factor for HNSCC, and TAR5 expression contributed to a longer OS time. Why does TAR5 have an opposing function profile between HNSCC and other cancers? This is a valuable question to be explored in the future. In addition, FTH1 (HR, 1.002), CHMP1A (HR, 1.01), and TYK2 (HR, 0.94) showed similar results in previous studies [28–30]. The rest of the genes have not been explored in HNSCC at present, so further research could concentrate on understanding the exact molecular mechanism of these genes. To verify the generality of this model, two independent GEO data sets (GSE65858 and GSE4163) were utilized to validate the effectiveness. Using the Kaplan–Meier curves of OS time and ROC curve analysis, similar results generated from GSE65858 and GSE4163 proved the feasibility of our prognostic model.

In addition, we made use of GSEA to explore the enrichment ways and characteristics in the two risk groups. Several metabolisms and mitochondrion-related pathways were found to play indispensable roles in the TCGA cohort, indicating that the metabolic phenotype may be significantly altered during necroptosis. In addition, mitochondria, the center of energy supply and biosynthesis, maintain basic cell survival, and dysfunctional mitochondria usually lead to cell death [31–33]. When mitochondria are impaired, numerous reactive oxygen species (ROS) are released, and the effects of increased ROS have been identified as an essential driver of cancer cell necroptosis [34, 35]. Previous studies have revealed that ROS, stimulated by tumor necrosis factor, can stabilize the necroptosis complex to induce cell necroptosis via promoting RIPK1 autophosphorylation and recruiting RIPK3 [36, 37]. Moreover, in colon cancer cells, by cooperating with RIPK1/RIPK3, ROS can facilitate cytosolic calcium accumulation and give rise to striking necroptosis [38]. Similarly, Basit et al. confirmed that, in melanoma cells, increased mitophagy-dependent ROS, caused by mitochondrial complex I inhibition, could also trigger necroptosis [35]. Taken together, according to these conclusions, it is undoubtedly a fact that mitochondrial ROS is essential for necroptosis, and the enhanced mitochondrion activity, as well as metabolism processes in our results, may be greatly associated with the elevated ROS level in HNSCC.

Finally, to validate our results, we utilized various online databases to analyze the seven NRGs involved in our model. The TIMER database was used to check the differential expression of NRGs, HPA was utilized to analyze the protein expression of NRGs in different tissues, and the cBioPortal database analyses were performed to explore gene alterations. Across these analyses, we obtained similar results, which suggested that the dysfunctional regulatory pathways, not the gene mutation, may be the main reasons for the alterations in NRG expression. Future explorations could focus on the regulatory pathways related to these seven NRGs to clarify the detailed necroptosis mechanism in HNSCC.

## 5. Conclusions

Collectively, we established a model of necroptosis-related signature with excellent ability to predict the OS time of HNSCC patients and validated it by two independent GEO data sets (GSE65858, GSE4163). Furthermore, we provided some new insights into the relationship between necroptosis and HNSCC. Future studies should continue to examine the validity of our model in order to applicate it in the clinical diagnosis one day.

## Figures and Tables

**Table 1:** Multivariate Cox results of NRGs based on TCGA-HNSCC.

Id	coef	HR	HR.95L	HR.95H	*p* value
TRAF5	−0.31157	0.732297	0.528807	1.014091	0.060693
PPID	0.094121	1.098693	1.047821	1.152036	9.98*E* − 05
VDAC1	0.009295	1.009338	1.002487	1.016236	0.007476
FTH1	0.002142	1.002145	1.001099	1.003192	5.79*E* − 05
CHMP3	0.054985	1.056525	1.010606	1.104531	0.015295
CHMP1A	0.009986	1.010036	0.997459	1.022771	0.118306
TYK2	−0.06185	0.940028	0.899074	0.982847	0.006504

Note: coef: coefficient; NRG: necroptosis-related genes; HR: hazard ratio.

## Data Availability

The data we used to analyze in this article are accessible by the public databases: The Cancer Genome Atlas (TCGA) databases and Gene Expression Omnibus (GEO) database (accession numbers: GSE65858 and GSE4163).

## Supplementary Materials

Supplementary Materials Figure S1: the mutation of the all DE-NRGs. Figure S2: the correlation between single NRGs and clinicopathological parameters. Table S1: necroptosis-related genes. Table S2: the results of PPI analysis. Table S3: univariate Cox results of NRGs based on TCGA-HNSCC. Tables S4–S5: KEGG enrichment results of high- and low-risk groups via GSEA. Tables S6–S7: GO enrichment results of high- and low- risk groups via GSEA.

## References

[1] Galluzzi L., Bravo-San Pedro J. M., Vitale I. (2015). Essential versus accessory aspects of cell death: recommendations of the NCCD 2015. *Cell Death and Differentiation*.

[2] Green D. R., Galluzzi L., Kroemer G. (2014). Cell biology. Metabolic control of cell death. *Science*.

[3] Conrad M., Angeli J. P. F., Vandenabeele P., Stockwell B. R. (2016). Regulated necrosis: disease relevance and therapeutic opportunities. *Nature Reviews Drug Discovery*.

[4] Cao L., Mu W. (2021). Necrostatin-1 and necroptosis inhibition: pathophysiology and therapeutic implications. *Pharmacological Research*.

[5] Berghe T. V., Linkermann A., Jouan-Lanhouet S., Walczak H., Vandenabeele P. (2014). Regulated necrosis: the expanding network of non-apoptotic cell death pathways. *Nature Reviews Molecular Cell Biology*.

[6] Khoury M. K., Gupta K., Franco S. R., Liu B. (2020). Necroptosis in the pathophysiology of disease. *The American Journal of Pathology*.

[7] Galluzzi L., Vitale I., Abrams J. M. (2012). Molecular definitions of cell death subroutines: recommendations of the nomenclature committee on cell death 2012. *Cell Death & Differentiation*.

[8] Vandenabeele P., Declercq W., Van Herreweghe F., Vanden Berghe T. (2010). The role of the kinases RIP1 and RIP3 in TNF-induced necrosis. *Science Signaling*.

[9] Tang R., Xu J., Zhang B. (2020). Ferroptosis, necroptosis, and pyroptosis in anticancer immunity. *Journal of Hematology & Oncology*.

[10] Feng X., Song Q., Yu A., Tang H., Peng Z., Wang X. (2015). Receptor-interacting protein kinase 3 is a predictor of survival and plays a tumor suppressive role in colorectal cancer. *Neoplasma*.

[11] Koo G.-B., Morgan M. J., Lee D.-G. (2015). Methylation-dependent loss of RIP3 expression in cancer represses programmed necrosis in response to chemotherapeutics. *Cell Research*.

[12] Strilic B., Yang L., Albarrán-Juárez J. (2016). Tumour-cell-induced endothelial cell necroptosis via death receptor 6 promotes metastasis. *Nature*.

[13] Ando Y., Ohuchida K., Otsubo Y. (2020). Necroptosis in pancreatic cancer promotes cancer cell migration and invasion by release of CXCL5. *PLoS One*.

[14] Bray F., Ferlay J., Soerjomataram I., Siegel R. L., Torre L. A., Jemal A. (2018). Global cancer statistics 2018: GLOBOCAN estimates of incidence and mortality worldwide for 36 cancers in 185 countries. *CA: A Cancer Journal for Clinicians*.

[15] Lee Y. C. A., Li S., Chen Y. (2019). Tobacco smoking, alcohol drinking, betel quid chewing, and the risk of head and neck cancer in an East Asian population. *Head & Neck*.

[16] Johnson D. E., Burtness B., Leemans C. R., Lui V. W. Y., Bauman J. E., Grandis J. R. (2020). Head and neck squamous cell carcinoma. *Nature Reviews Disease Primers*.

[17] Leemans C. R., Braakhuis B. J. M., Brakenhoff R. H. (2011). The molecular biology of head and neck cancer. *Nature Reviews Cancer*.

[18] Pollheimer M. J., Kornprat P., Lindtner R. A. (2010). Tumor necrosis is a new promising prognostic factor in colorectal cancer. *Human Pathology*.

[19] Li J., Huang S., Zeng L. (2020). Necroptosis in head and neck squamous cell carcinoma: characterization of clinicopathological relevance and in vitro cell model. *Cell Death & Disease*.

[20] Karki R., Sharma B. R., Tuladhar S. (2021). Synergism of TNF-*α* and IFN-*γ* triggers inflammatory cell death, tissue damage, and mortality in SARS-CoV-2 infection and cytokine shock syndromes. *Cell*.

[21] Pasparakis M., Vandenabeele P. (2015). Necroptosis and its role in inflammation. *Nature*.

[22] Aaes T. L., Kaczmarek A., Delvaeye T. (2016). Vaccination with necroptotic cancer cells induces efficient anti-tumor immunity. *Cell Reports*.

[23] Ou D., Garberis I., Adam J. (2018). Prognostic value of tissue necrosis, hypoxia-related markers and correlation with HPV status in head and neck cancer patients treated with bio- or chemo-radiotherapy. *Radiotherapy and Oncology*.

[24] Hodgson A., Xu B., Satkunasivam R., Downes M. R. (2018). Tumour front inflammation and necrosis are independent prognostic predictors in high-grade urothelial carcinoma of the bladder. *Journal of Clinical Pathology*.

[25] Sun G., Zheng C., Deng Z., Huang C., Huang J. (2020). TRAF5 promotes the occurrence and development of colon cancer via the activation of PI3K/AKT/NF-kappaB signaling pathways. *Journal of Biological Regulators and Homeostatic Agents*.

[26] Liang Z., Li X., Liu S., Li C., Wang X., Xing J. (2019). MiR-141-3p inhibits cell proliferation, migration and invasion by targeting TRAF5 in colorectal cancer. *Biochemical and Biophysical Research Communications*.

[27] Ma Y., Duan J., Hao X. (2020). Down‐regulated HDAC3 elevates microRNA‐495‐3p to restrain epithelial‐mesenchymal transition and oncogenicity of melanoma cells via reducing TRAF5. *Journal of Cellular and Molecular Medicine*.

[28] Hu Z.-W., Wen Y.-H., Ma R.-Q. (2021). Ferroptosis driver SOCS1 and suppressor FTH1 independently correlate with M1 and M2 macrophage infiltration in head and neck squamous cell carcinoma. *Frontiers in Cell and Developmental Biology*.

[29] Wintergerst L., Selmansberger M., Maihoefer C. (2018). A prognostic mRNA expression signature of four 16q24.3 genes in radio (chemo) therapy‐treated head and neck squamous cell carcinoma (HNSCC). *Molecular Oncology*.

[30] Fang L., Wang W., Shi L., Chen Q., Rao X. (2021). Prognostic values and clinical relationship of TYK2 in laryngeal squamous cell cancer. *Medicine*.

[31] Tait S. W. G., Oberst A., Quarato G. (2013). Widespread mitochondrial depletion via mitophagy does not compromise necroptosis. *Cell Reports*.

[32] Bock F. J., Tait S. W. G. (2020). Mitochondria as multifaceted regulators of cell death. *Nature Reviews Molecular Cell Biology*.

[33] Gaschler M. M., Hu F., Feng H., Linkermann A., Min W., Stockwell B. R. (2018). Determination of the subcellular localization and mechanism of action of ferrostatins in suppressing ferroptosis. *ACS Chemical Biology*.

[34] Moloney J. N., Cotter T. G. (2018). ROS signalling in the biology of cancer. *Seminars in Cell & Developmental Biology*.

[35] Basit F., van Oppen L. M., Schöckel L. (2017). Mitochondrial complex I inhibition triggers a mitophagy-dependent ROS increase leading to necroptosis and ferroptosis in melanoma cells. *Cell Death & Disease*.

[36] Zhang Y., Su S. S., Zhao S. (2017). RIP1 autophosphorylation is promoted by mitochondrial ROS and is essential for RIP3 recruitment into necrosome. *Nature Communications*.

[37] Schenk B., Fulda S. (2015). Reactive oxygen species regulate Smac mimetic/TNF*α*-induced necroptotic signaling and cell death. *Oncogene*.

[38] Sun W., Wu X., Gao H. (2017). Cytosolic calcium mediates RIP1/RIP3 complex-dependent necroptosis through JNK activation and mitochondrial ROS production in human colon cancer cells. *Free Radical Biology and Medicine*.

