# Supplementary material for: Development and Validation of a Necroptosis-Related Prognostic Model in Head and Neck Squamous Cell Carcinoma
Source: J Oncol. 2022 Feb 18;2022:8402568. doi: 10.1155/2022/8402568 (PMC8881120; doi:10.1155/2022/8402568)
Supplement: Supplementary Materials — Figure S1: the mutation of the all DE-NRGs. Figure S2: the correlation between single NRGs and clinicopathological parameters. Table S1: necroptosis-related genes. Table S2: the results of PPI analysis. Table S3: univariate Cox results of NRGs based on TCGA-HNSCC. Tables S4–S5: KEGG enrichment results of high- and low-risk groups via GSEA. Tables S6–S7: GO enrichment results of high- and low- risk groups via GSEA. [file 8402568.f1.zip › 8402568.f1/Supplemental Figre S1 (1).pdf]

Supplement Figure 1

A

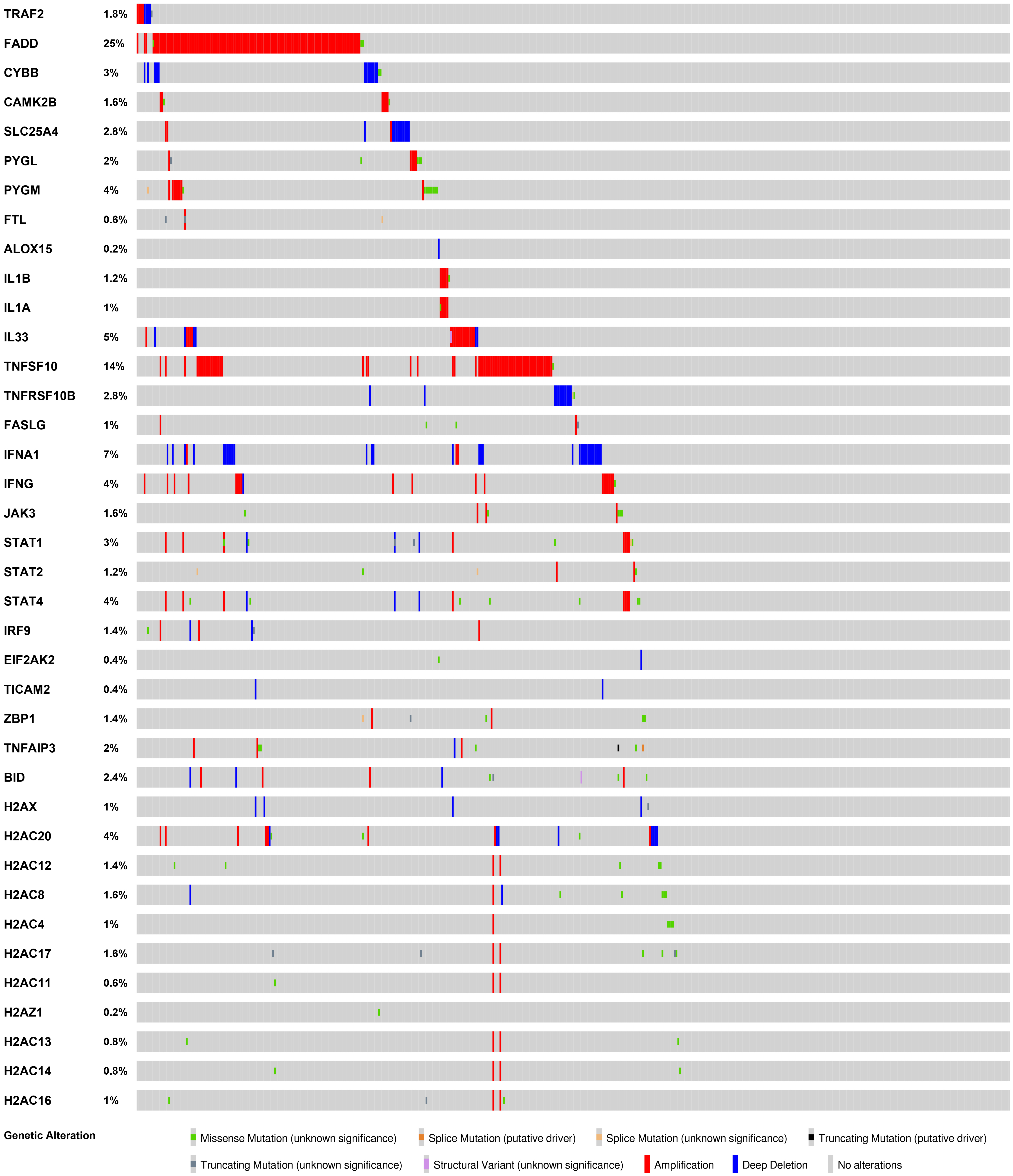

Figure S1: Mutation of the all differentially expressed NRGs

(A): The gene alteration of 38 DE-NRGs. “Amplification” and “Deep Deletion” were the main types of genetic alteration.
