# Supplementary material for: Development and Validation of a Necroptosis-Related Prognostic Model in Head and Neck Squamous Cell Carcinoma
Source: J Oncol. 2022 Feb 18;2022:8402568. doi: 10.1155/2022/8402568 (PMC8881120; doi:10.1155/2022/8402568)
Supplement: Supplementary Materials — Figure S1: the mutation of the all DE-NRGs. Figure S2: the correlation between single NRGs and clinicopathological parameters. Table S1: necroptosis-related genes. Table S2: the results of PPI analysis. Table S3: univariate Cox results of NRGs based on TCGA-HNSCC. Tables S4–S5: KEGG enrichment results of high- and low-risk groups via GSEA. Tables S6–S7: GO enrichment results of high- and low- risk groups via GSEA. [file 8402568.f1.zip › 8402568.f1/Supplemental Figre S2 (1).pdf]

Supplement Figure 2

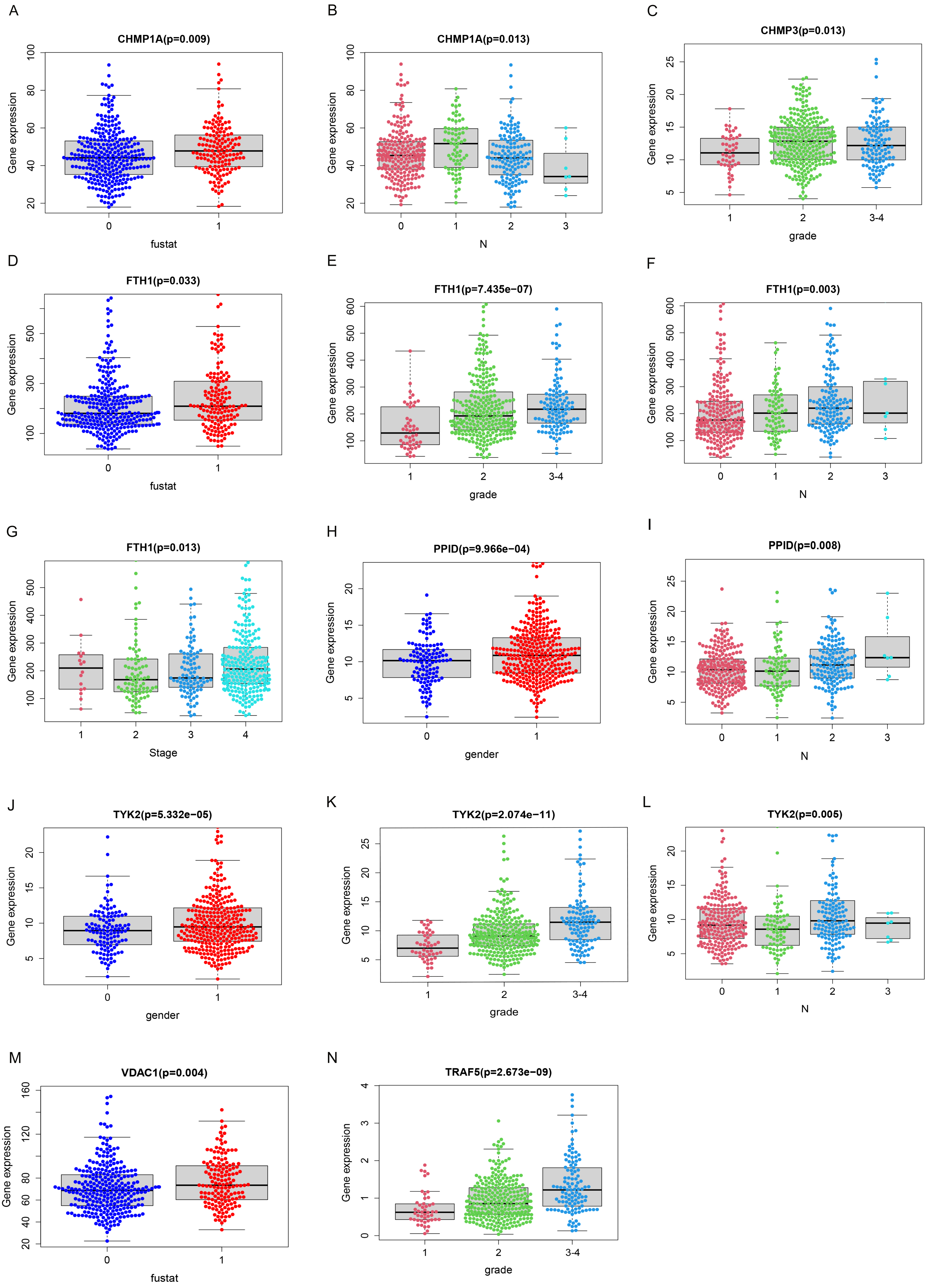

Figure S2: correlation between single NRGs and clinicopathological parameters.

(A)-(B): The association between CHMP1A and survival outcome, tumor N stage. (C): The association between CHMP3 and tumor grade. (D)-(G): The association between FTH1 and survival ourcome, tumor grade, tumor N stage, tumor stage. (H)-(I): The association between PPID and gender, tumor N stage. (J)-(L): The association between TYK2 and geneder, tumor grade, tumor N stage. (M): The association between VDAC1 and survival outcome. (N): The association between TRAF5 and tumor grade.
